# TLR4 and RB1 as the Identified Lactate‐Related Genes to Predict the Diagnostic Performance, Gene Regulatory Network, and Targeting Drugs in Depression

**DOI:** 10.1155/humu/5401795

**Published:** 2026-05-30

**Authors:** Fengmin Ding, Jianbei Chen, Jiajia Wu, Qinglai Bian

**Affiliations:** ^1^ School of Basic Medical Sciences, Hubei University of Chinese Medicine, Wuhan, China, hbtcm.edu.cn; ^2^ School of Traditional Chinese Medicine, Hubei University of Chinese Medicine, Wuhan, China, hbtcm.edu.cn

**Keywords:** depression, drugs, lactate, molecular docking, random forest, RB transcriptional corepressor 1, Toll-like receptor 4

## Abstract

**Background:**

Lactate is implicated in several brain diseases; however, its precise role in depression remains to be further elucidated. The current study looks into the role of lactate‐associated genes in depression, along with their diagnostic and therapeutic potential.

**Methods:**

Genes related to lactate were obtained from the Harmonizome database. Depression‐related datasets were acquired from the GEO database, and differentially expressed genes (DEGs) were identified using the limma package. The overlapping genes from the DEGs and lactate‐related genes were subjected to enrichment analyses using enrichR. The correlation analysis confirmed the validation of screened core genes via the random forest algorithm. Cytoscape was used for the construction of transcription factors and miRNA‐based gene regulatory networks. Receiver operating characteristics (ROC) analysis was used to evaluate diagnostic performance. Molecular docking was performed to predict drug interactions.

**Results:**

The lactate‐related DEGs were seen to be implicated in processes like negative regulation of the apoptotic process and NOD‐like receptor signaling pathways. TLR4 and RB1 were identified as core genes, showing elevated expression and strong diagnostic potential in depression. In addition to the positive correlation, TLR4 was shown to be the target of multiple miRNAs, while RB1 was unveiled to be the target of several transcription factors. Besides, the binding of RB1 (protein: 6C2R) to topotecan and dexamethasone was confirmed, while TLR4 (protein: 4R7N) could bind with Tlr4‐IN‐C34 and resatorvid.

**Conclusion:**

This study successfully identified TLR4 and RB1 as core lactate–related genes in depression, providing a new perspective on elucidating the role of lactate in the pathogenesis of depression.

## 1. Introduction

Depression refers to a common, costly, and debilitating condition related to an increased suicide risk, which has also become one of the leading causes of public health issues across the globe and affects over 300 million people, while the number is still increasing [1–4]. Traditional antidepressant pharmacotherapies concentrate on enhancing the monoaminergic tone; however, as to the efficacy, such drugs, consistently, demonstrate a delayed response in spite of their acute effects on the monoaminergic system [5–7]. In addition to the lack of well‐defined biological mechanisms, there remains a gap between the number of people suffering from depression and the capacity to provide effective treatment that leads to remission, underscoring the urgent need for a transformation in therapeutic approaches [8, 9].

Both microarray technology and bioinformatics have already been extensively applied in genetic testing to identify the relevant differentially expressed genes (DEGs) and functional pathways related to the diagnosis of depression [10–12]. Moreover, bioinformatics analyses have been applied to unravel biomarkers and signaling pathways correlated with depression [13]. For instance, an existing study has unveiled seven mitochondria‐related diagnostic biomarkers for depression, which were also correlated with the immune infiltrating cells [14]. Another study based on eight prefrontal cortex‐related datasets ffrom the Gene Expression Omnibus (GEO) revealed seven hub DEGs with downregulated expression levels as potential diagnostic biomarkers for depression [15]. Further multiomics data applied in Mendelian randomization analysis have led to the identification of potential pathogenic oxidative stress‐related genes in depression, with four genes further recognized as influencing disease risk through methylation and expression alterations [16]. This evidence, collectively, prompts us to unveil additional relevant diagnostic biomarkers for future therapeutic exploration.

Lactate, which may be the best known metabolic waste product, was initially isolated from sour milk and primarily known as a crucial signaling molecule continuously forming under aerobic conditions [17–19]. Moreover, some prior evidence has demonstrated the role of lactate as an important energy source as well as a metabolic signaling molecule for the brain, where it may be involved in development, synaptic plasticity, angiogenesis, and even disease [20]. While researchers have been trying to elucidate the role of lactate in depression, it has also been demonstrated that hypocretin‐1/hypocretin receptor 1 may regulate hippocampal lactate homeostasis to regulate neuroplasticity and cognitive function in a depressed model induced by chronic unpredictable mild stress (CUMS) [21]. Additionally, lactate may trigger the emergence of depression‐like behaviors caused by blue light exposure during sleep [22]. Conversely, the role of lactate as an antidepressant has also been addressed, which mediates resilience to stress and may be associated with the modulation of the hippocampal levels and activity of histone deacetylases (HDACs) [23]. Such evidence has been supported by a recent discovery unveiling that the acute administration of lactate may exert an antidepressant‐like effect via cAMP‐dependent protein synthesis [24]. Although existing evidence reveals the multifaceted role of lactate in depression, its specific regulatory mechanisms remain unclear, and systematic studies on related genes in depression are still limited. Therefore, further investigation into the role of lactate and its associated genes in depression holds significant theoretical value and clinical importance. In this study, based on the data from publicly available databases like GEO, we aimed to identify some lactate‐related gene biomarkers and their potential targeting drugs using both computational analyses and molecular docking. We hope that the results of the present study can provide additional evidence regarding the role of lactate in depression.

## 2. Methods

**Figure 1:**
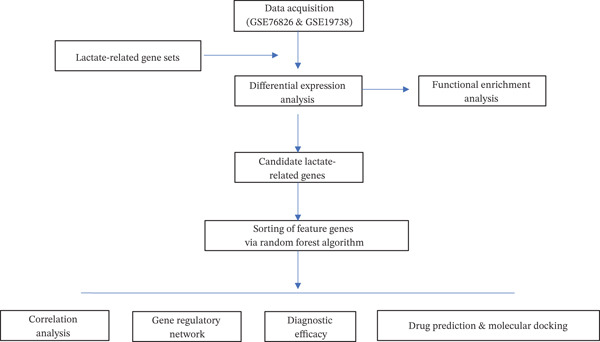
Flowchart describing the analytical procedures on the lactate‐related genes in depression. The depression‐associated datasets (GSE19738 and GSE76826) and lactate‐related gene sets were collected for the differential expression analysis. Then, the obtained genes were summarized for both functional enrichment analysis and sorting of feature genes via the random forest algorithm. Thereafter, the feature genes identified were applied for the correlation analysis, the plotting of gene regulatory network, and the prediction of diagnostic efficacy and relevant targeting drugs (with the latter further validated by molecular docking).

The whole analytical procedure of the current study is shown in Figure 1.

### 2.1. Data Source

A list of 327 lactate‐related genes was collected from the Harmonizome Database 3.0 (https://maayanlab.cloud/Harmonizome/) using the keywords “lactic acid” and “lactate” [25]. The data of gene expression and clinical information relevant to depression were collected based on the datasets GSE19738 (132 cases, 66 normal vs. 66 diseases) and GSE76826 (32 cases, 12 normal vs. 20 diseases). The expression matrix and metadata matrix were extracted, then using the R package “GEOquery” (Version 2.58.0) [26].

### 2.2. Identification of Lactate‐Related DEGs

The following functions of the R package “limma” (Version 3.46.0), including “normalizeBetweenArrays,” “lmFit,” and “eBayes” were applied to identify the DEGs at the thresholds *p* value < 0.05 (for the dataset GSE19738) and *p* value < 0.01 (for the dataset GSE76826). The relevant results of the DEGs were shown in the volcano plot, which was drawn using the R package “ggplot2” (Version 3.4.0) [27].

Thereafter, the obtained DEGs of the datasets GSE19738 and GSE76826 were intersected with the lactate‐related genes to further define the lactate‐related DEGs.

### 2.3. Functional Enrichment Analysis

The obtained lactate‐related DEGs of the aforementioned analyses were then applied for the functional enrichment analysis of Gene Ontology (GO) based on the three subcategories including cellular component, biological process, and molecular function and Kyoto Encyclopedia of Genes and Genomes (KEGG) via the R package “enrichR” (Version 3.1) [28].

### 2.4. Random Forest Analysis

The supervised machine learning algorithm random forest was applied to further characterize the core lactate–related genes in depression via the R package “randomForest” (Version 4.7‐1.1) [29]. The minimal number of the first 3000 trees with a tendency of flattened error rate was checked using the function “randomForest,” followed by the remodeling and the determination of “MeanDecreaseAccuracy” and “MeanDecreaseGini” via the function “varImpPlot.” The top 5 genes based on the “MeanDecreaseGini” were recognized as lactate‐related disease‐specific core genes and finally applied for the analysis.

### 2.5. Correlation Analysis

The correlation analysis on the aforementioned core genes was performed using the function “formatted_cors” of the R package “Corrgram” (Version 1.14), and Pearson’s coefficient was calculated, with values close to 1 or ‐1 indicating strong positive or negative correlation, respectively. The corresponding results were displayed in a heatmap.

### 2.6. Construction of Gene Regulatory Network

To elucidate the upstream regulatory mechanisms of the core genes, we identified potential transcription factors (TFs), microRNAs (miRNAs), and long noncoding RNAs (lncRNAs) using the R package “enrichR” (Version 3.1) and visualized the resulting gene regulatory network with Cytoscape (Version 3.9.1).

### 2.7. Construction of the Diagnostic Model

The expression levels of the core genes in diseased and control samples were first quantified based on the datasets GSE19738 and GSE76826. Then, the R package “ROCit” (Version 2.1.1) was applied to draw the receiver operating characteristics (ROC) curves via the output probability of the two classifications to calculate the area under the curve (AUC) values and the 90% confidence intervals (90% CIs) to determine the diagnostic potential of the core genes.

### 2.8. Molecular Docking

The list of FDA‐approved drugs and the 3D protein structure files of the core genes were downloaded from the ZINC database (https://zinc.docking.org/substances/subsets/) and the PDB database (https://www.rcsb.org/). The docking pocket of the proteins was determined via (1) the POCASA tool (Version 1.1) or (2) the residue base in the structure based on existing publications. The sorting of the drug candidates was accomplished in AutoDockVina, and the molecular structure was visualized in PyMOL.

### 2.9. Statistical Analyses

The computational analyses were performed in R software (Version 3.6.0), and the data of the two groups were compared using the Wilcoxon test. Pearson’s correlation analysis was applied for the correlation analysis. The overall statistical significance was determined when *p* value was lower than 0.05 (*p* value < 0.05).

## 3. Results

### 3.1. Identification of Lactate‐Related DEGs in Depression

**Figure 2:**
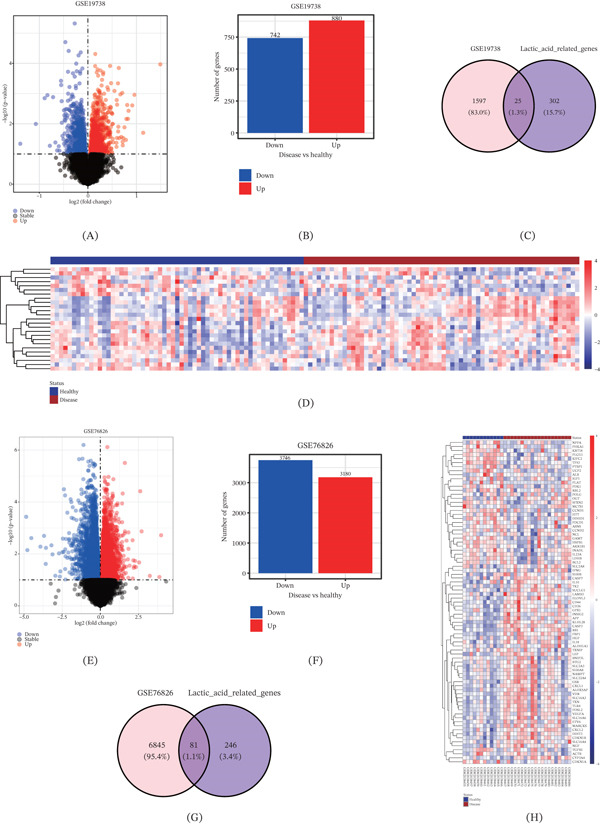
Screening of differentially expressed lactic acid–related genes in the datasets GSE19738 and GSE76826. (A) Volcano plot showing the differentially expressed genes in disease sample and healthy control of the dataset GSE19738. (B) Histogram showing the number of differentially expressed genes of the dataset GSE19738. (C) Venn diagram intersecting the differentially expressed genes of dataset GSE19738 and lactic acid–related genes. (D) The expression levels of the differentially expressed genes in all samples with the color indicating the *Z*‐score of gene expression. (E) Volcano plot showing the differentially expressed genes in disease sample and healthy control of the dataset GSE76826. (F) Histogram showing the number of differentially expressed genes of the dataset GSE76826. (G) Venn diagram intersecting the differentially expressed genes of dataset GSE76826 and lactic acid–related genes. (H) The expression levels of the differentially expressed genes in all samples with the color indicating the *Z*‐score of gene expression.

Differential analysis was performed on the two datasets GSE19738 and GSE76826 to compare the DEGs between disease samples and healthy control samples. According to the GSE19738 dataset, 880 upregulated and 742 downregulated DEGs were identified (Figure 2A,B), and there were 25 overlapping genes following the intersection of the DEGs with the lactate‐related genes (Figure 2C,D). In the GSE76826 dataset, 3180 upregulated and 3746 downregulated DEGs were seen (Figure 2E,F), and 81 overlapping genes were recognized after taking the intersection of DEGs with lactate–related genes (Figure 2G,H).

**Figure 3:**
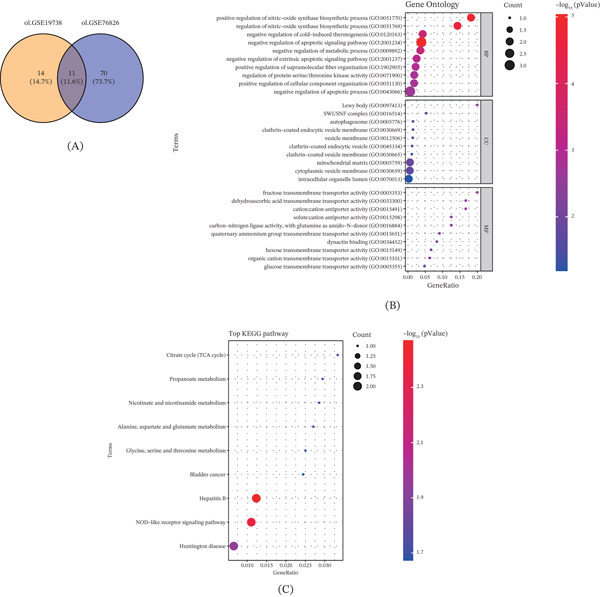
Screening of lactic acid–related feature genes in the datasets GSE19738 and GSE76826. (A) Venn diagram intersecting the lactic acid–related differentially expressed genes in the datasets GSE19738 and GSE76826. (B) Gene Ontology enrichment analysis (biological process [BP]; cellular component [CC]; molecular function [MF]) applied to unveil the top enriched pathways of the feature genes. (C) KEGG signaling pathway enrichment analysis showing the top enriched signaling pathways of the feature genes.

The overlapping genes from the datasets GSE19738 and GSE76826 were intersected again to reduce the number of lactate‐related feature genes for our analysis, yielding a list of 11 overlapping genes (Figure 3A). The results of GO and KEGG enrichment analyses have manifested the enrichment in processes like negative regulation of the apoptotic process and NOD‐like receptor signaling pathways, for instance (Figure 3B,C). These findings suggest that lactic acid–related genes may participate in the pathological mechanisms of depression by regulating the apoptotic process and NOD‐like receptor signaling pathways, providing crucial evidence for subsequent screening and functional analysis of core genes.

### 3.2. Identification of Lactate‐Related Core Genes in Depression

**Figure 4:**
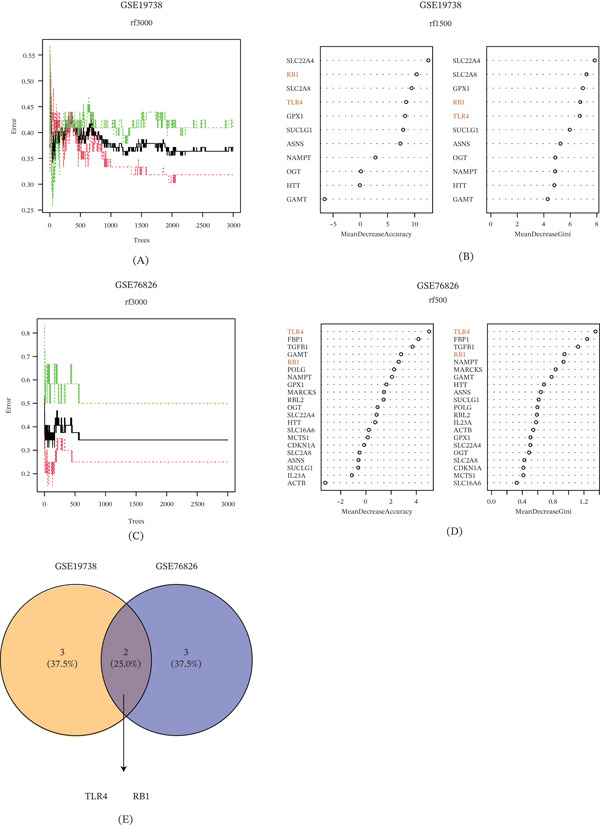
Screening for disease‐specific lactate‐related core genes in depression. (A, B) Random forest algorithm displaying the error rate distribution of 3000 trees and the potential candidate genes sorted by the MeanDecreaseAccuracy and MeanDecreaseGini values in the dataset GSE19738. (C, D) Random forest algorithm displaying the error rate distribution of 3000 trees and the potential candidate genes in the dataset GSE76826. (E) Intersection of the common genes from these datasets to reveal the core genes.

Thereafter, the supervised machine learning algorithm Random Rorest was applied to the datasets GSE19738 and GSE76826 to unveil the core genes, and then, the MeanDecreaseAccuracy and MeanDecreaseGini values of the interested genes were calculated based on the sorting results using the “varImpPlot” function (Figure 4A–D and Tables S1 and S2). The overlapping genes of the two datasets following the analysis were intersected and taken as the core genes, which were Toll‐like receptor 4 (TLR4) and RB transcriptional corepressor 1 (RB1) (Figure 4E).

### 3.3. Correlation Analysis and the Gene Regulatory Network of the Lactate‐Related Core Genes in Depression

**Figure 5:**
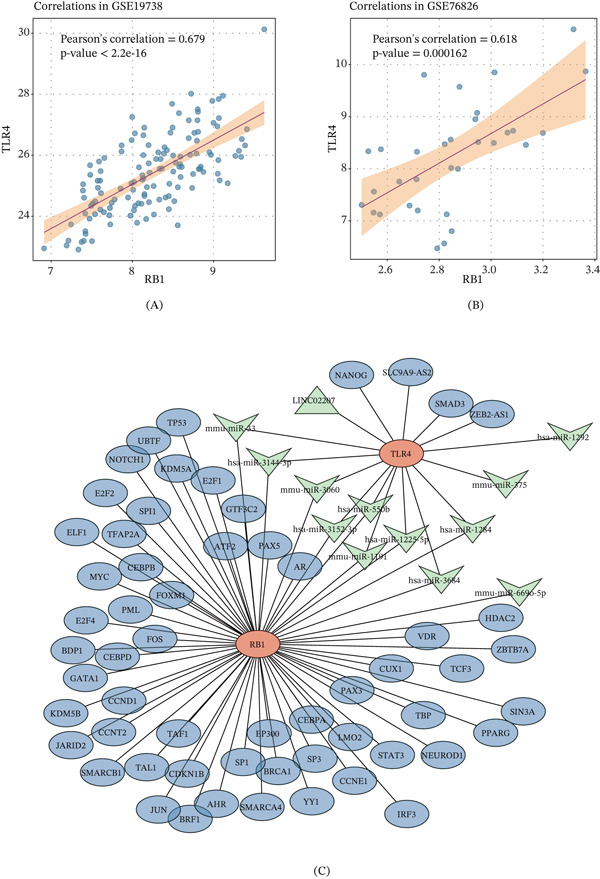
Correlation analysis and the gene regulatory network of the lactate‐related core genes in depression. (A, B) Correlation analysis on the core genes TLR4 and RB1 in the datasets GSE19738 and GSE76826. (C) The gene regulatory network plotted based on the core genes TLR4 and RB1 (in orange). The green nodes represent the potential targeted miRNAs/lncRNAs, and the blue nodes are the transcription factors.

The correlation of these two core genes TLR4 and RB1 in the datasets GSE19738 and GSE76826 was further explored, and a positive correlation between these two core genes was visible (Figure 5A,B). In the meantime, the gene regulatory network involving these two core genes as well as their potential targeted TFs, miRNAs, and lncRNAs was plotted, where TLR4 was shown to be the target of multiple miRNAs, while RB1 was unveiled to be the target of several TFs (Figure 5C). These findings suggest that TLR4 and RB1 exert synergistic effects through the regulation of molecules at different levels, indicating their potential joint involvement in complex transcriptional regulatory networks during the onset and progression of depression.

### 3.4. Diagnostic Performance of the Core Lactate–Related Genes in Depression

**Figure 6:**
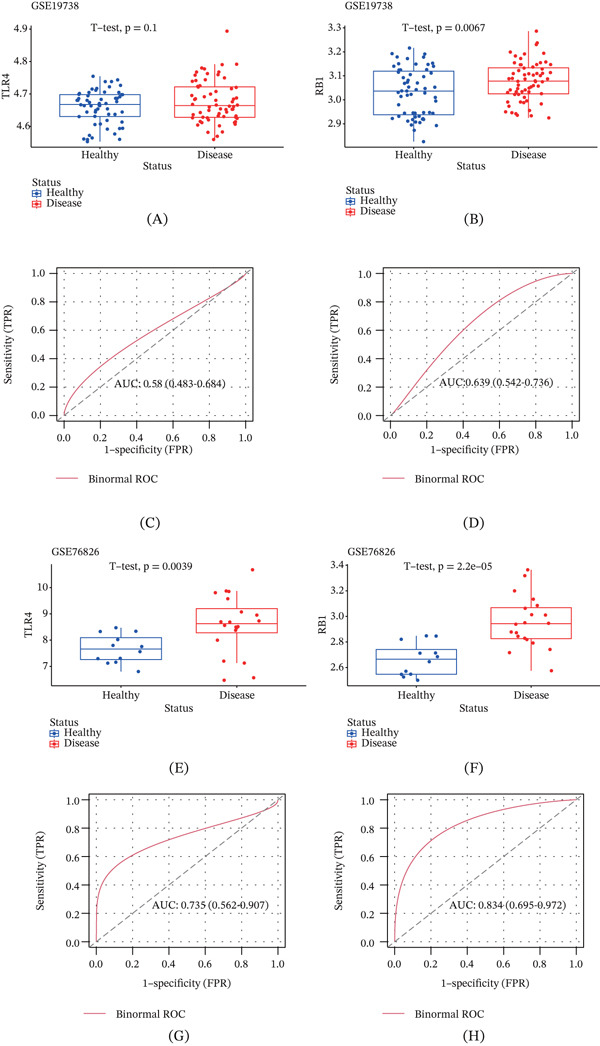
Diagnostic performance of the core lactate–related genes in depression. (A, B) Box plot showing the expression levels of the core genes TLR4 and RB1 in the dataset GSE19738. (C, D) The binormal ROC curve showing the diagnostic performance of the core genes TLR4 and RB1 in the dataset GSE19738 (the AUC value and 90% confidence interval were also calculated). (E, F) Box plot showing the expression levels of the feature genes TLR4 and RB1 in the dataset GSE76826. (G, H) The binormal ROC curve showing the diagnostic performance of the feature genes TLR4 and RB1 in the dataset GSE76826 (the AUC value and 90% confidence interval were also calculated).

In addition, we aimed to explore the diagnostic potential of the core genes in depression, and their expression levels in diseased samples and healthy controls were determined. In the dataset GSE19738, the expression levels of TLR4 and RB1 were higher in diseased samples than those of healthy controls (Figure 6A,B). The AUC values for TLR4 and RB1 were 0.58 (90% CI: 0.483–0.684) and 0.639 (90% CI: 0.542–0.736), respectively, indicating their diagnostic potential (Figure 6C,D). As to the dataset GSE76826, the expression levels of TLR4 and RB1 were also seen to be higher in diseased samples (Figure 6E,F), and the strong diagnostic potential of these two genes was supported by the AUC of 0.735 (90% CI: 0.562–0.907) and 0.834 (90% CI: 0.695–0.972) (Figure 6G,H). These results indicate that TLR4 and RB1 exhibit consistent upregulation trends in two independent datasets and demonstrate strong diagnostic efficacy, suggesting their potential as stable and promising diagnostic biomarkers for depression.

### 3.5. Molecular Docking on the Predicted Drugs and the Core Lactate–Related Genes

**Figure 7:**
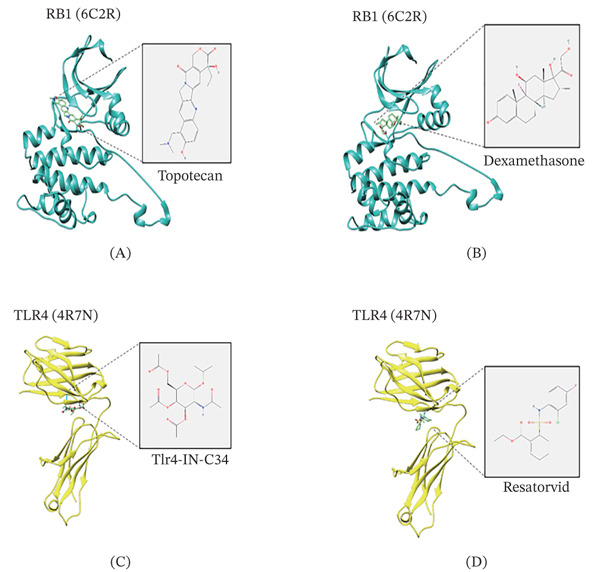
Molecular docking on the predicted drugs and the core lactate–related genes. (A, B) Molecular docking showing the binding between the core gene RB1 (protein structure 6C2R) and the targeted drugs topotecan or dexamethasone. (C, D) Molecular docking showing the binding between the core gene TLR4 (protein structure 4R7N) and the targeted drugs Tlr4‐IN‐C34 or resatorvid.

In accordance with the data of molecular docking analysis, the binding of RB1 (protein: 6C2R) to topotecan and dexamethasone was confirmed (Figure 7A,B), while TLR4 (protein: 4R7N) could bind with Tlr4‐IN‐C34 and resatorvid (Figure 7C,D).

## 4. Discussion

Modern medicine has contributed to increasing knowledge with regard to the pathophysiology of mental disorders [30, 31]; however, some problems persist when it comes to the modern clinical diagnostics of depression, which has prompted the application of diagnostics and taxonomy of different types of depression using modern molecular biomarkers [32–36]. This study used bioinformatics analysis and molecular docking technology to identify for the first time the two core lactate–related genes in depression, which are TLR4 and RB1. The results showed that these two genes were significantly upregulated in depressed patients, had excellent diagnostic performance, and were both synergistically regulated by TFs and miRNAs. Molecular docking further demonstrated that RB1 could bind to topotecan and dexamethasone, while TLR4 could bind to Tlr4‐IN‐C34 and resatorvid, providing novel drug candidates for targeted therapy in depression. This research not only enhances the mechanistic understanding of the action of lactate‐associated genes in depressive disorders but also provides a theoretical foundation and experimental basis for the development of diagnostic biomarkers and drug screening of depression.

Lactate has been referred to as the final product of glycolysis, which features not only as an energy substrate, a metabolite, and a signaling molecule but also as a modulator of protein lactylation (a novel epigenetic modification considered to be critical for energy metabolism and signaling in brain tissues) [37]. However, so far as we are concerned, it remains to be further explored whether lactate‐related genes could be applied as the therapeutic targets of depression. Existing studies, mostly, discussed the role of lactate‐related genes in determining the survival, immune cell infiltration, and response to immunotherapies in the context of oncology [38–40]. Functional enrichment analysis revealed that the overlapping lactate‐related DEGs were primarily enriched in pathways such as negative regulation of apoptotic process and NOD‐like receptor signaling pathways. The role of apoptosis, which is generally regarded to be necessary for the prevention of cancerous growth, in depression has already been interpreted, as exemplified by the discovery suggesting that chronic stress (which can precipitate depression) increases the susceptibility of certain neuronal populations to cell death [41]. Also, the increased levels of caspase‐3 active (a known apoptosis promoter) have been seen in patients with major depressive disorder [42]. The NOD‐like receptors are critical to our health, and notably, NOD‐like receptor 3 (NLRP3) has been underscored as a key player in the pathogenesis of depression [43, 44]. Such evidence re‐emphasized the roles of apoptotic process and NOD‐like receptor signaling pathways in depression and paved the way for subsequent identification of relevant genes which may modulate these two processes.

Then, using the random forest algorithm, TLR4 and RB1 were ultimately identified as the core lactate–related genes in depression. TLR4, a transmembrane receptor involved in the innate immune response, not only engages with the exogenous ligands on the surface of the cellular membrane but also interacts with the intracellular ligands to initiate the intracellular signaling cascades, which play a regulatory role in behavioral despair induced by chronic social defeat stress[45, 46]. RB1 is the first described tumor‐suppressor gene previously known to play an integral role in the development of both retinoblastoma and some tumor diseases [47, 48]. While trying to link these two core genes with depression, it has been documented that levomilnacipran may ameliorate lipopolysaccharide (LPS)‐induced depression‐like behaviors in rats via targeting TLR4 and that TLR4/NLRP3 axis may participate in the effects of Xiaoyansan against depression [49, 50]. Certainly, lactate has been shown to suppress TLR4‐mediated inflammation through GPR81‐mediated inhibition of NF‐*κ*B activation. We further hypothesize that lactate may modulate TLR4‐driven neuroinflammation in depression through this mechanism which may contribute to a positive feedback injury loop wherein glycolysis induced by inflammation further sustains TLR4 signaling [51, 52]. In addition, emerging evidence suggests that RB1 may influence lactate metabolism by modulating the expression of lactate transporters or glycolytic enzymes [53]. The combination of the TLR4 and RB1 could be viewed as two distinct but related links between lactate metabolism and depression. The first one is the link between lactate signaling and neuroinflammation. The second one is lactate usage and cellular energy homeostasis.

In our present study, following the confirmation that TLR4 and RB1 could be the core lactate–related genes in depression and that TLR4 and RB1 were positively correlated with each other, a strong diagnostic potential of these two genes was seen, and the results of molecular docking suggested that RB1 could bind with topotecan and dexamethasone, while TLR4 could bind with Tlr4‐IN‐C34 and resatorvid. Of the identified targeted drugs, the efficacy of dexamethasone has been addressed, although it has been suggested that its repeated administration to LPS‐inflamed mice may trigger the onset of depression‐like behaviors [54]. As to the remaining drug candidates, topotecan is a topoisomerase 1 inhibitor which is known to exert tumor‐suppressive activity via stabilizing the enzyme‐mediated DNA cleavage complex [55, 56]. Tlr4‐IN‐C34 is a 2‐acetamidopyranoside which may partially repress the conduction of the TLR4 signaling pathway, while resatorvid is another known TLR4‐specific inhibitor with anti‐inflammatory properties [57, 58]. While the specific mechanisms of these compounds on depression need additional laboratory and clinical verification, our current study preliminarily explored their potential in the treatment of depression, thereby providing evidence for follow‐up studies.

Nonetheless, despite our attempt to reveal the involvement of lactate‐related genes and their potential targeting drugs in depression, some shortcomings need to be addressed in future studies. For instance, we utilized a set of lactate‐related genes for analysis, which could have excluded other potential key genes associated with depression but not yet classified in the lactate pathway. Future studies could make use of unbiased genome‐wide expression data complemented by meta‐analysis to comprehensively reveal core molecular biomarkers for depression. Second, our findings are entirely based on retrospective data from public databases with relatively limited sample sizes and lack independent external validation cohorts. Further research should incorporate multicenter, large‐sample clinical cohorts for prospective validation to enhance the generalizability and reliability of the results. Thirdly, the molecular docking results only provide computational predictions of the binding affinities of core genes and candidate drugs without functional validation in vitro or in vivo. Future studies should use cellular models and animal models of depression to pharmacologically validate the targeting of TLR4 and RB1 so as to clarify their therapeutic effects in depression. Finally, the substantial time span between the two datasets may introduce technical heterogeneity and potential batch effects; subsequent analyses should apply more rigorous batch correction algorithms or select more recent and homogeneous datasets for replication validation. To conclude, the present study provides novel insights into the lactate‐related mechanisms of depression. However, these limitations need to be addressed in future studies through more systematic experimental designs and multidimensionally validated.

## 5. Conclusion

Collectively speaking, this study identifies TLR4 and RB1 as core lactate–related genes in depression, demonstrating their consistent upregulation and diagnostic potential across independent cohorts. Functional enrichment analysis links these genes to apoptosis regulation and NOD‐like receptor signaling. The findings suggest possible connections between lactate metabolism and immune‐related pathways in depression. Future work concerning the regulatory networks involving TFs and miRNAs will generate testable hypotheses regarding how these genes function. From a translational standpoint, the predicted binding of drugs to TLR4 and RB1 provides a foundation for experimental validation in preclinical models, although their therapeutic significance remains to be determined.

**Nomenclature**
DEGs: differentially expressed genes
GEO: Gene Expression Omnibus
CUMS: chronic unpredictable mild stress
HDACs: histone deacetylates
GO: Gene Ontology
KEGG: Kyoto Encyclopedia of Genes and Genomes
TFs: transcription factors
miRNAs: microRNAs
lncRNAs: long noncoding RNAs
ROC: receiver operating characteristics
AUC: area under the curve
90% CIs: 90% confidence intervals
TLR4: Toll‐like receptor 4
RB1: RB transcriptional corepressor 1
NLRP3: NOD‐like receptor 3
LPS: lipopolysaccharide

## Author Contributions

All authors contributed to this present work. Fengmin Ding and Qinglai Bian designed the study. Jiajia Wu acquired the data. Qinglai Bian interpreted the data. Jianbei Chen drafted the manuscript. Fengmin Ding revised the manuscript.

## Funding

The study was funded by Guiding Scientific Research Program of Hubei Provincial Department of Education (B2022120), Joint supported by Hubei Provincial Natural Science Foundation and Innovation and Development of Traditional Chinese Medicine of China (2023AFD134 and 2026AFC0911), Postdoctoral Fellowship Program (Grade C) of China Postdoctoral Science Foundation (GZC20252613), and China Postdoctoral Science Foundation (2025M773932).

## Disclosure

All authors read and approved the manuscript.

## Ethics Statement

Ethical approval was not required for this study because it did not involve any human or animal experiments.

## Consent

The authors have nothing to report.

## Conflicts of Interest

The authors declare no conflicts of interest.

## Supporting Information

Additional supporting information can be found online in the Supporting Information section.

## Supporting information


**Supporting Information 1** Table S1: Random forest algorithm on identifying the feature genes in the dataset GSE19738.

**Supporting Information 2** Table S2: Random forest algorithm on identifying the feature genes in the dataset GSE76826.

## Data Availability

The datasets generated and/or analyzed during the current study are available in the GSE19738 repository (https://www.ncbi.nlm.nih.gov/geo/query/acc.cgi?acc=GSE19738) and GSE76826 repository (https://www.ncbi.nlm.nih.gov/geo/query/acc.cgi?acc=GSE76826).
