# Supplementary material for: TLR4 and RB1 as the Identified Lactate‐Related Genes to Predict the Diagnostic Performance, Gene Regulatory Network, and Targeting Drugs in Depression
Source: Hum Mutat. 2026 May 30;2026:5401795. doi: 10.1155/humu/5401795 (PMC13239030; doi:10.1155/humu/5401795)
Supplement: Supplementary file 1 — Supporting Information 1 Table S1: Random forest algorithm on identifying the feature genes in the dataset GSE19738. [file HUMU-2026-5401795-s001.docx]

**Supplementary Table 1. Random forest algorithm on identifying the feature genes in the dataset GSE19738**

|  | Disease | Healthy | MeanDecreaseAccuracy | MeanDecreaseGini |
| --- | --- | --- | --- | --- |
| ACTB | 0.306136 | 0.599628 | 0.568149 | 1.652041 |
| ASNS | -0.51735 | 6.695918 | 4.898167 | 2.798347 |
| CDKN1A | -5.37726 | 4.735719 | -0.27533 | 2.238827 |
| FBP1 | 1.524306 | 5.037853 | 5.205782 | 3.661314 |
| GAMT | -3.65796 | 0.130151 | -2.27289 | 2.234622 |
| GPX1 | 3.532739 | 4.880986 | 5.625172 | 4.007652 |
| HTT | -0.38587 | -0.59222 | -0.46642 | 2.666987 |
| IL23A | -1.96654 | 6.814754 | 3.392296 | 3.310889 |
| MARCKS | -2.06022 | 2.68459 | 0.557388 | 2.50519 |
| MCTS1 | 3.189163 | 3.325517 | 4.665412 | 3.513744 |
| NAMPT | 1.813434 | 1.07889 | 2.011505 | 2.600036 |
| OGT | -1.72776 | 1.877663 | 0.130408 | 2.669434 |
| POLG | -0.61593 | 1.950305 | 1.06433 | 2.932734 |
| RB1 | 3.257327 | 9.758814 | 9.148358 | 3.871065 |
| RBL2 | -1.92637 | 3.591413 | 1.613794 | 2.353315 |
| SLC16A6 | 4.721003 | 6.438494 | 7.569701 | 3.792996 |
| SLC22A4 | 4.920966 | 5.15453 | 7.00898 | 4.144035 |
| SLC2A8 | 0.587647 | 10.52494 | 8.122547 | 4.610306 |
| SUCLG1 | 3.559419 | 3.381833 | 4.940465 | 3.406295 |
| TGFB1 | 3.592035 | -1.66054 | 1.415614 | 2.797276 |
| TLR4 | 5.618702 | 2.471023 | 5.674435 | 3.722723 |
