# Supplementary material for: TLR4 and RB1 as the Identified Lactate‐Related Genes to Predict the Diagnostic Performance, Gene Regulatory Network, and Targeting Drugs in Depression
Source: Hum Mutat. 2026 May 30;2026:5401795. doi: 10.1155/humu/5401795 (PMC13239030; doi:10.1155/humu/5401795)
Supplement: Supplementary file 2 — Supporting Information 2 Table S2: Random forest algorithm on identifying the feature genes in the dataset GSE76826. [file HUMU-2026-5401795-s002.docx]

**Supplementary Table 2. Random forest algorithm on identifying the feature genes in the dataset GSE76826**

|  | Disease | Healthy | MeanDecreaseAccuracy | MeanDecreaseGini |
| --- | --- | --- | --- | --- |
| ACTB | -0.48496 | -3.15767 | -3.22629 | 0.542323 |
| ASNS | -1.21794 | 0.435628 | -0.58497 | 0.640198 |
| CDKN1A | 0.286874 | -0.21388 | -0.13785 | 0.413819 |
| FBP1 | 3.192207 | 3.638859 | 4.16911 | 1.242478 |
| GAMT | 1.162768 | 3.750094 | 2.788388 | 0.77914 |
| GPX1 | 0.652018 | 1.170806 | 1.627487 | 0.50585 |
| HTT | 2.175235 | -0.61664 | 0.728999 | 0.680193 |
| IL23A | 0.262478 | -1.93088 | -1.11477 | 0.577994 |
| MARCKS | 0.490324 | 2.447986 | 1.432439 | 0.832569 |
| MCTS1 | 1.168767 | -0.83236 | 0.149103 | 0.412695 |
| NAMPT | 1.921663 | 1.23976 | 2.071647 | 0.9342 |
| OGT | 1.329026 | -0.01493 | 0.923125 | 0.487742 |
| POLG | 2.846345 | 0.158133 | 2.234499 | 0.595359 |
| RB1 | 1.671302 | 2.612142 | 2.605517 | 0.947357 |
| RBL2 | 1.846266 | 0.615132 | 1.400846 | 0.58856 |
| SLC16A6 | 0.596697 | -0.30162 | 0.231109 | 0.326158 |
| SLC22A4 | 0.033672 | 0.968834 | 0.85029 | 0.504163 |
| SLC2A8 | 0.663494 | -1.36284 | -0.50841 | 0.425429 |
| SUCLG1 | -0.59815 | 0.248865 | -0.61139 | 0.611415 |
| TGFB1 | 2.946107 | 2.47776 | 3.691317 | 1.123847 |
| TLR4 | 2.869294 | 5.100655 | 5.020874 | 1.350137 |
